# Relaxins enhance growth of spontaneous murine breast cancers as well as metastatic colonization of the brain

**DOI:** 10.1007/s10585-013-9609-2

**Published:** 2013-08-21

**Authors:** Claudia Binder, Eugenia Chuang, Christina Habla, Annalen Bleckmann, Matthias Schulz, Ross Bathgate, Almuth Einspanier

**Affiliations:** 1 Department of Haematology/Oncology, Georg-August-University, Robert-Koch-str. 40, 37075 Göttingen, Germany; 2 Institute of Veterinary Physiological Chemistry, University of Leipzig, An den Tierkliniken 1, 04103 Leipzig, Germany; 3 Deparment of Medical Statistics, Georg-August-University, Göttingen, Germany; 4 Florey Neuroscience Institutes and Department of Biochemistry and Molecular Biology, University of Melbourne, Parkville, VIC Australia

**Keywords:** Breast cancer, Brain metastasis, Tg(MMTV-erbB2) mouse model, Relaxin, RXFP1

## Abstract

Relaxins are known for their tissue remodeling capacity which is also a hallmark of cancer progression. However, their role in the latter context is still unclear, particularly in breast cancer. In a mouse model with spontaneously arising breast cancer due to erbB2-overexpression we show that exposure to porcine relaxin results in significantly enhanced tumour growth as compared to control animals. This is accompanied by increased serum concentrations of progesterone and estradiol as well as elevated expression of the respective receptors and the relaxin receptor RXFP1 in the tumour tissue. It is also associated with enhanced infiltration by tumour-associated macrophages which are known to promote tumour progression. Additionally, we show in an ex vivo model of metastatic brain colonization that porcine relaxin as well as human brain-specific relaxin-3 promotes invasion into the brain tissue and enhance interaction of breast cancer cells with the resident brain macrophages, the microglia. Relaxin signaling is mediated via RXFP1, since R 3/I5, a specific agonist of the relaxin-3 receptor RXFP3 in the brain, does not significantly enhance invasion. Taken together, these findings strongly support a role of relaxins in the progression of breast cancer where they foster primary tumour growth as well as metastatic colonization by direct and indirect means.

**Electronic supplementary material:**

The online version of this article (doi:10.1007/s10585-013-9609-2) contains supplementary material, which is available to authorized users.

## Introduction

The mammalian peptide hormone relaxin and its human analogue H2 relaxin or relaxin-2 are well known for their matrix-modifying capacity. They induce extensive tissue remodeling via upregulation of matrix metalloproteinases (MMP) and angiogenesis [1, 2]. Since this is also a key feature of malignant invasion, they have been implicated in cancer progression. However, their role in this context is still unclear.

The most unambiguous data exist for prostate cancer. In xenografts of relaxin-2-overexpressing prostate cancer cell lines, tumour growth and neoangiogenesis are significantly enhanced [3]. The opposite can be observed after transfection of an antagonistic relaxin-2 analogue lacking the binding domain for the relaxin receptor RXFP1 [4]. Increased production of relaxin-2 as well as relaxin by tumour and stromal cells has been demonstrated in advanced prostate cancers in humans and mice [5, 6]. Relaxin enhances proliferation, adhesion and invasion in vitro as well as tumour growth in the TRAMP mouse model in vivo, while deficiency of RXFP1 antagonizes this effect [5, 7]. A tumour-promoting function of relaxin-2 has been demonstrated also in thyroid cancer where it fosters invasion in vitro via increased invadopodia formation and MMP upregulation as well as xenograft growth in nude mice [8, 9]. Relaxin-2 expression was also enhanced in advanced human endometrium cancers correlating with unfavourable clinical outcome [10].

In breast cancer, the situation is much less clear. Elevated expression of relaxin-2 has been demonstrated in neoplastic mammary tissues as compared to their benign counterparts [11]. Porcine relaxin as well as relaxin-2 was found to influence tumour cell proliferation in a biphasic way regarding time course and concentration. While low amounts and short-term application resulted in enhanced in vitro growth of MCF-7 and MDA MB-231 cells, high concentrations and long-term exposure yielded the opposite effect and diminished the growth of the respective xenografts in nude mice [12–14]. We showed that 5-day exposure of MCF-7 and SK-BR3 cells to 100 ng/ml of porcine relaxin as well as recombinant relaxin-2 was followed by MMP upregulation and enhanced invasiveness. This could be inhibited by the antagonistic relaxin-2 analogue B-R13/17 K [15, 16]. Consistently, relaxin-2 serum levels were significantly elevated in patients with metastatic breast cancer correlating with short survival [17].

To further complicate matters, relaxins do not only act on the tumour cells but also on the benign cells of the surrounding stromal compartment. Components of the tumour microenvironment are essentially involved in malignant progression, in particular, the tumour-associated macrophages (TAM) which are characterized by a so-called M2 phenotype with tumour-promoting function [18]. We have recently shown that not only the TAM, infiltrating from the peripheral blood, but also resident macrophages at the site of metastasis are critical for the colonization of distant organs [19, 20]. Interestingly, Figueiredo et al. [21] have shown that relaxin inhibits expression of the typical M1-cytokine IL 1β in rat macrophages, thus indicating a potential role for relaxins in the tumour-associated phenotype shift to M2.

Given the impact of microenvironment effects on tumour growth we searched for a model where the influence of relaxins on breast cancer progression could be studied without confounding factors, such as xenografting, artificial cancer induction/injection or immunodeficiency. We therefore chose the Tg(MMTV-erbB2) mouse model, where breast cancers arise spontaneously due to transgenic erbB2-overexpression [22]. Since erbB2-overexpressing tumours are clinically aggressive and often metastasize into the brain [23], we additionally used the organotypic brain slice coculture, an ex vivo model recently established by our group [19], to study the effect of porcine relaxin as well as the human brain isoform relaxin-3 on the colonization of the central nervous system.

## Materials and methods

### Animals and experimental design

Animal experiments were approved by the local committee of Animal Care and Use (nr. 509.42502/01-37.01). Tg(MMTV-erbB2) mice (Charles River, Sulzfeld, Germany) were housed and bred under standard conditions. Hemizygous female mice develop spontaneous cancers in almost all mammary glands within 4–6 months after birth due to overexpression of the rat erbB2-transgene driven by the MMTV-promotor [22]. Presence of the transgene was confirmed by DNA analysis in 45 female mice, which were then divided into a control (*n* = 23) and a relaxin group (*n* = 22). From day 100 on after birth animals were monitored closely by daily inspection and palpation. Osmotic pumps (nr. 2004, Alzet, Cupertino/Canada), filled either with porcine relaxin (6.5 ng/ml; 0.28 μl/h) or 0.9 % sodium chloride, were implanted into the neck area at the first sign of mammary gland tumour development (d 1 of the study period). After euthanasia, mammary glands were examined for tumour development. Tumours were measured and weighed before fixation in liquid nitrogen and paraformaldehyde. Internal organs were inspected for metastasis and were collected for histological evaluation.

### Determination of serum relaxin, estradiol and progesterone

Serum relaxin concentrations were determined using an enzyme immunoassay which had been validated for porcine and human relaxin as described previously [24]. Since our aim was primarily to detect porcine relaxin in order to confirm the correct delivery via the pumps, this assay was suitable. The lower detection limit was 0.07 ng/ml. It has been shown earlier that the antibody used for this assay also detects murine relaxin. To confirm this, we performed measurements in six pregnant mice (day 17) yielding values corresponding to the literature for this stage of pregnancy. Upon serial dilution, the curve was parallel to our porcine standard curve. As to be expected, relaxin was undetectable in 6 male mice. Serum estrogen (E2) levels were examined using a commercial ELISA kit (IBL International GmbH, Hamburg, Germany) according to the manufacturer’s instructions. Serum progesterone (P4) was measured via enzyme immunoassay as described previously [24]. The lower detection limits were 0.016 ng/ml for E2 and 0.06 ng/ml for P4, respectively.

### Histology and immunohistochemistry

Organs were embedded in paraffin, cut (5 μm sections, microtome HM 36, Microm, Walldorf, Germany) and mounted on 0.01 % poly-l-lysine coated glass slides (Sigma, Deisenhofen, Germany). Hematoxylin-eosin staining (HE; Merck, Darmstadt, Germany) was performed for morphological overview. For immunohistochemical analysis, the following primary antibodies were used: monoclonal rabbit anti estrogen receptor (ER) α (Euromedex, Souffel Weyersheim, France, 1:1,000), rabbit anti progesterone receptor (PR) (Immunotech, Krefeld, Germany, 1:4,000), polyclonal rabbit anti porcine relaxin (serum 258, courtesy of OD Sherwood, Illinois, US), mouse anti human clone MAC 387 (Dako, Hamburg, Germany, 1:600) for the histiocyte antigen MAC 387; polyclonal rabbit anti mouse Ki67 (1:400, courtesy of Dr. Scholzen, Jülich, Germany) and polyclonal rabbit anti human RXFP1 (1:2,000, courtesy of Richard Ivell, Dummerstorf, Germany). The validation protocol for the RXFP1 antibody has been described in [25]. To ensure that it also recognizes the murine receptor we additionally validated the antibody in mouse uterine and heart tissue. This yielded the same results as to be expected from the literature. After deparaffinization, pre-treatment with citrate solution (pH 6, 40 min at 90 °C) and a trypsin digestion step (15 min), slides were incubated with 3 % H_2_O_2_ (20 min) for inhibition of endogenous peroxidases. The primary antibodies were applied at 4 °C overnight. Stain was developed using horseradish peroxidase-coupled goat anti-mouse/anti-rabbit secondary antibodies (DAKO EnVision detection kit, Dako Cytomation, Hamburg, Germany) and the Vector AEC substrate kit as a chromogen (Linaris, Germany). Mouse-anti-rabbit-IgG (Dianova, Hamburg, Germany) was used as negative control. Microscopical evaluation was performed independently by two different persons counting 100 cells per sample and classifying them as positive or negative.

### RT-PCR

Total RNA was extracted with the guanidinium thiocyanate method [26] followed by reverse transcription using oligo-dT primers (Invitrogen GmbH, Karlsruhe, Germany). The murine RXFP1 transcript as well as human RXFP1,2 and 3 were analyzed by semi-quantitative RT-PCR using ribosomal 18 and 26S as controls. Primers were designed using the NCBI Primer-BLAST (http://ncbi.nlm.nih.gov/tools/primer-blast/index.cgi) based on the published sequence [27, 28]. RT-PCR was performed on the RotorGene 6000 (Corbett Lifescience, Qiagen, Hilden, Germany) with the 2× SensiMix dt Kit (Quantace Ltd, London, UK) according to the instructions of the manufacturer. Each sample was analyzed in duplicate and in two separate runs. The PCR cycle conditions for the primers were as follows: 10 min at 95 °C, 45 cycles of 30 s at 54–58 °C, 15 s at 72 °C with a final melting curve analysis to confirm the specifity of the amplified products. PCR products were analysed by gel electrophoresis.

### Organotypic brain slice coculture model

Slice cocultures were performed as described previously [19]. Briefly, NMRI mice (p5–p7) were decapitated, and brains were removed under aseptic conditions. Horizontal whole brain sections (400 μm, vibratome Leica VT1000S; Leica, Wetzlar, Germany) were transferred onto a 0.4 μm polycarbonate membrane in a transwell tissue insert (Falcon, model 3090, Becton–Dickinson) and incubated within a 6-well dish in 50 % MEM, 25 % Hanks’ balanced salt solution (Gibco, Karlsruhe, Germany), 25 % normal horse serum, 0.2 mM glutamine, 100 U/ml penicillin, 100 mg/ml streptomycin and 4.5 mg/ml glucose. After 24 h, 10^5^ tumour cells, embedded in 20 μl RPMI with 85 % extracellular matrix gel (R&D Systems, Wiesbaden, Germany) were placed next to the intact outside of the slice. The breast cancer cell lines used for these experiments were either transfected with a GFP-expression vector or stained with the fluorescent membrane dye PKH67 (Sigma-Aldrich, Steinheim, Germany). Porcine relaxin, synthetic human relaxin-3 and the RXFP3-agonist R3/I5 were added to the medium at the indicated concentrations. After 72 h, brain slices and tumour plug were fixated in 4 % paraformaldehyde, removed together from the inserts and washed with PBS/0.2 % Triton-X-100. Slices and adherent tumour plugs were then stained with fluorescence dye-conjugated *Griffonia simplicifolia* isolectin B4 (1:100, Alexa Fluor 568- ILB4; Invitrogen, Karlsruhe, Germany), mounted in DAKO fluorescent mounting medium (S-3023, DakoCytomation, Glostrup, Denmark) and analyzed using a confocal laser scanning microscope (LSM 510, Zeiss, Göttingen, Germany).

### Statistics

Statistical analysis was carried out using the SPSS15 software (SPSS, Munich, Germany) and the free statistical software R (version 2.13.1; http://www.r-project.org). The influence of volume, day and treatment was investigated by using a multivariate linear regression model for both endpoints volume and weight. In order to assess the influence of the treatment, the slope of the regression lines of the two groups were compared. Parametric data were analysed with the two-sided Student’s *t* test. A *p* value <0.05 was considered significant.

## Results

### Relaxin enhances local tumour growth

The first palpable tumours developed between days 130 and 185 after birth predominantly in the caudal mammary glands with progression to almost all glands later on. After implantation of the osmotic pumps (day 1 of the experimental period), containing either porcine relaxin or sodium chloride, animals were monitored clinically and sacrificed out of ethical considerations in any case where tumour progression affected their well-being. Some of them were killed also at earlier time points after pump implantation to allow continuous assessment of tumour growth.

**Fig. 1:**
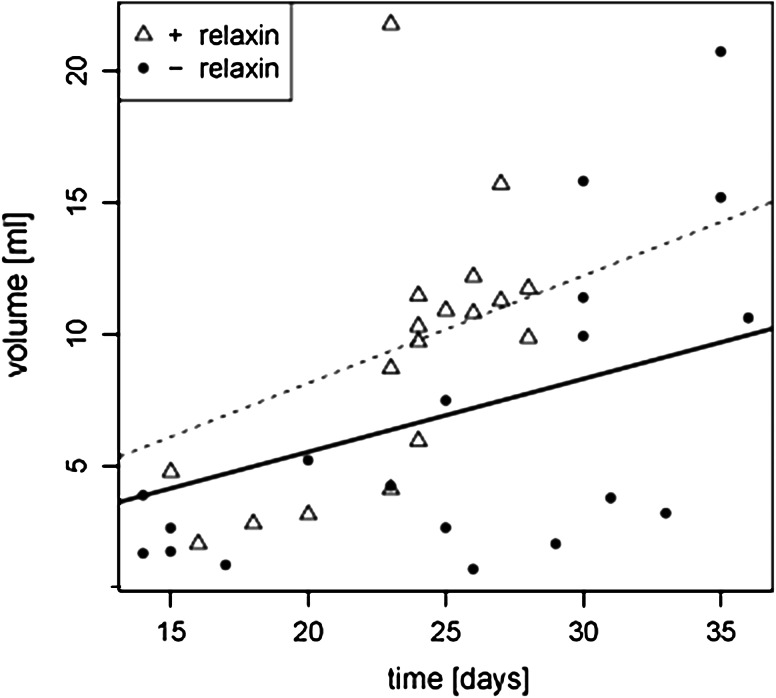
Porcine relaxin fosters local tumour growth in the Tg(MMTV-erbB2) mouse. Graphical presentation of the multivariate linear model of the volume–time correlation, showing earlier development of large tumours in animals treated with porcine relaxin than in controls (*p* = 0.033; *dashed line* linear regression curve relaxin group, *solid line* controls)

The median time to euthanasia was shorter in the relaxin group (Table 1). None of these animals could be left alive after day 29 of the experimental phase, in contrast to the control group where some animals lived until day 36. However, due to the elective early euthanasias the difference did not reach statistical significance. Evaluation of the total tumour volume per mouse confirmed that relaxin-treated mice developed larger tumours at earlier time points. The median total tumour volume was higher in relaxin-treated animals (Table 1). This was strongly significant considering the first 29 days where animals from both groups were still alive. Large tumours were found also in control animals, but only later on. Statistical analysis of the volume–time correlation using a multivariate linear model further corroborated these findings and yielded a significant difference in local tumour progression with faster growth in the relaxin-treated group (Fig. 1). The mean absolute body weight at euthanasia did not differ significantly (+ relaxin: 29 ± 2.1 g; controls: 30.6 ± 3.1 g; *p* = 0.11). There were no detectable metastases in any of the animals, neither macroscopically nor after histological evaluation of the potential target organs including the brain.

**Table 1:** Day of euthanasia (after pump implantation) and tumour volume

	+ Relaxin			Controls			*p* value
	Median	25–75 % quartiles	Min–max	Median	25–75 % quartiles	Min–max	
Total tumour volume (cm^3^)							
Days 1–29	10.1	4.7–11.4	2.1–21.8	2.6	1.7–4.2	1.1–7.5	0.0005
Days 1–36	10.1	4.7–11.4	2.1–21.8	3.9	2.2–10.4	1.1–20.7	>0.05
Day of euthanasia	24	23–26	15–28	27.5	18.5–32	14–36	>0.05

To confirm correct relaxin delivery, relaxin serum levels were determined at euthanasia (Table 2). As expected, we found significantly higher peripheral relaxin concentrations in the relaxin group than in the control animals. Moreover, serum estradiol (E2) and progesterone (P4) levels were also significantly increased in the relaxin group, thus, indicating an influence of relaxin on production of these hormones.

**Table 2:** Hormone serum concentrations at euthanasia (mean ± SD)

	+ Relaxin	Controls	*p* value
Relaxin (ng/ml)	0.71 ± 0.38	0.108 ± 0.109	0.000001
P4 (ng/ml)	0.79 ± 0.37	0.28 ± 0.22	0.000031
E2 (ng/ml)	0.23 ± 0.12	0.13 ± 0.52	0.0056

### Relaxin induces proliferation and facilitates macrophage invasion

**Fig. 2:**
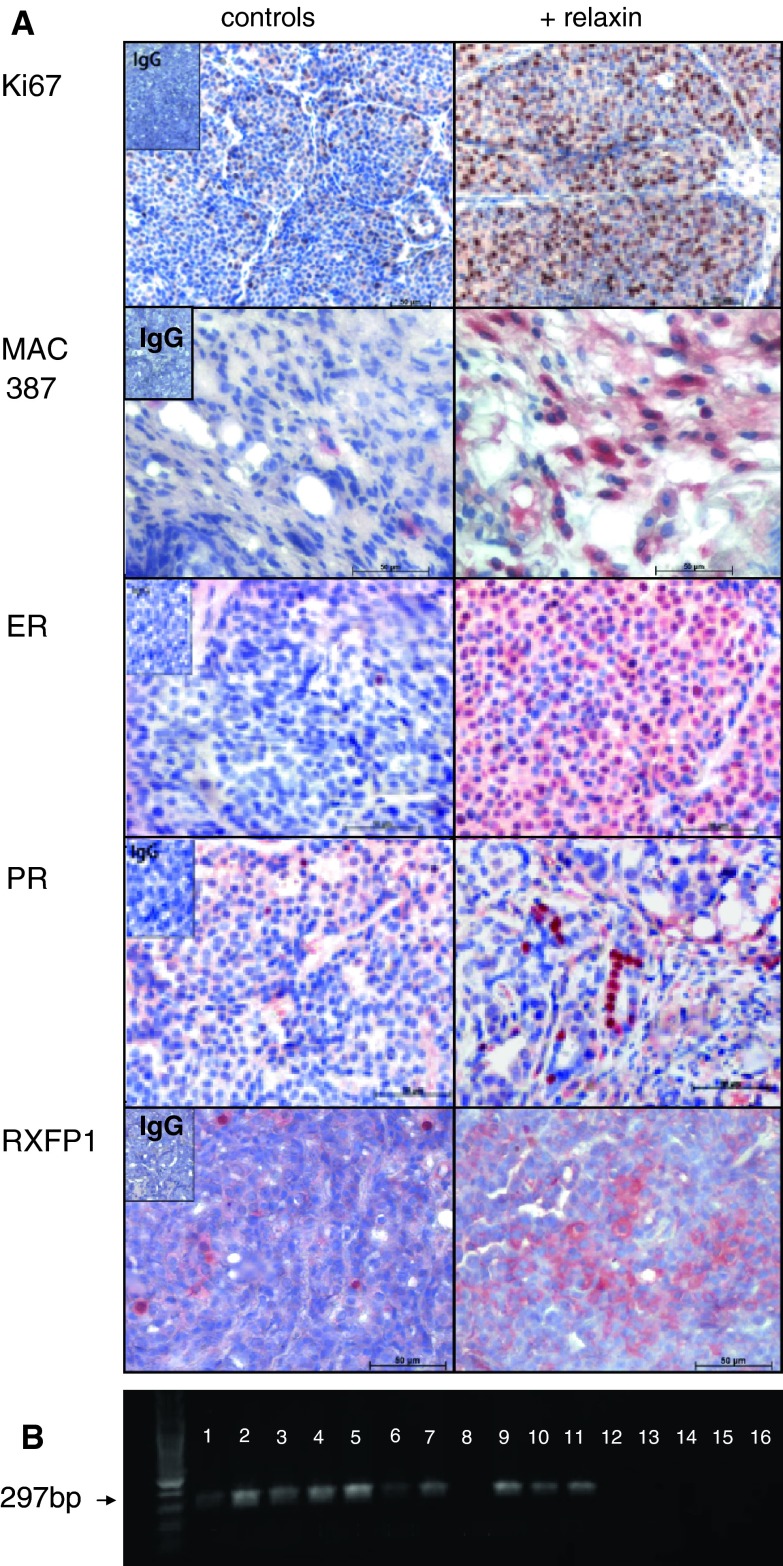
Porcine relaxin influences histological tumour characteristics. **a** Immunohistochemical assessment of proliferation (Ki67), macrophage infiltration (MAC 387), expression of hormone receptors and RXFP1, showing enhanced positivity for all parameters in the relaxin group. IgG negative controls are shown in inserts. *Scale bars* 50 μm, magnification ×40 (except Ki 67: ×20). **b** Expression of RXFP1 mRNA (RT-PCR) in tumours from relaxin-treated animals (*lane* *2*–*5*, *7*, *9*–*11*) but not in control animals (*lane* *1*, *6*, *8*, *12*–*15*; *lane* *16* neg. control)

Upon morphological examination of the H&E-stained samples, tumours in relaxin-treated and control animals did not differ from each other. There was no fibrous capsule around the tumours in either of the groups. However, there was a significant increase in the proliferation rate of tumours from relaxin-treated animals as shown by Ki67-staining (Table 3; Fig. 2). Expression of the ER was moderate with considerable interindividual variations in both groups, however, it was significantly lower in the control group. PR expression was generally low, but also significantly lower in the control tumours. Equally, RXFP1 was less frequently expressed in control tumours than in tumours from relaxin-treated mice (*p* < 0.01). Since macrophages are responsive to relaxin [21], we measured the amount of TAM in the various tumour samples. In fact, there was a significantly higher degree of TAM infiltration in the relaxin-exposed tumours than in the controls. TAM were not only localized in the stromal compartment as presented in Fig. 2, but also within the malignant tissues. The tumours also expressed relaxin, ranging from focal up to diffuse positivity, however, without any difference between control and relaxin group (suppl. Figure 1).

**Table 3:** Histological tumour characteristics (mean ± SD)

	+ Relaxin	Controls	*p* value
Ki67+ (%)	40.4 ± 11.05	8.18 ± 5.8	0.00000003
ER+ (%)	33.73 ± 11.05	15.90 ± 6.04	0.000064
PR+ (%)	6.2 ± 3.10	1.92 ± 1.16	0.00018
RXFP1+ (%)	39.2 ± 14.14	9.09 ± 5.2	0.00001
MAC 387+ (%)	38.8 ± 11.76	9.45 ± 4.27	0.0000005

### Relaxins enhance malignant colonization of brain tissue

In humans, erbB2-positive tumours tend to metastasize early and often disseminate into the brain. However, we were not able to detect any metastases. The animals had to be euthanized when the local tumours became too large, a time span which may have been too short to allow metastatic dissemination. To further address this question, we used a murine ex vivo model of brain colonization, recently established by our group [19]. There, invasion of tumour cells into the living brain tissue can be followed over several days. Additionally, the system allows observation of the reaction of stromal components, in particular the microglia, the resident macrophages of the brain.

**Fig. 3:**
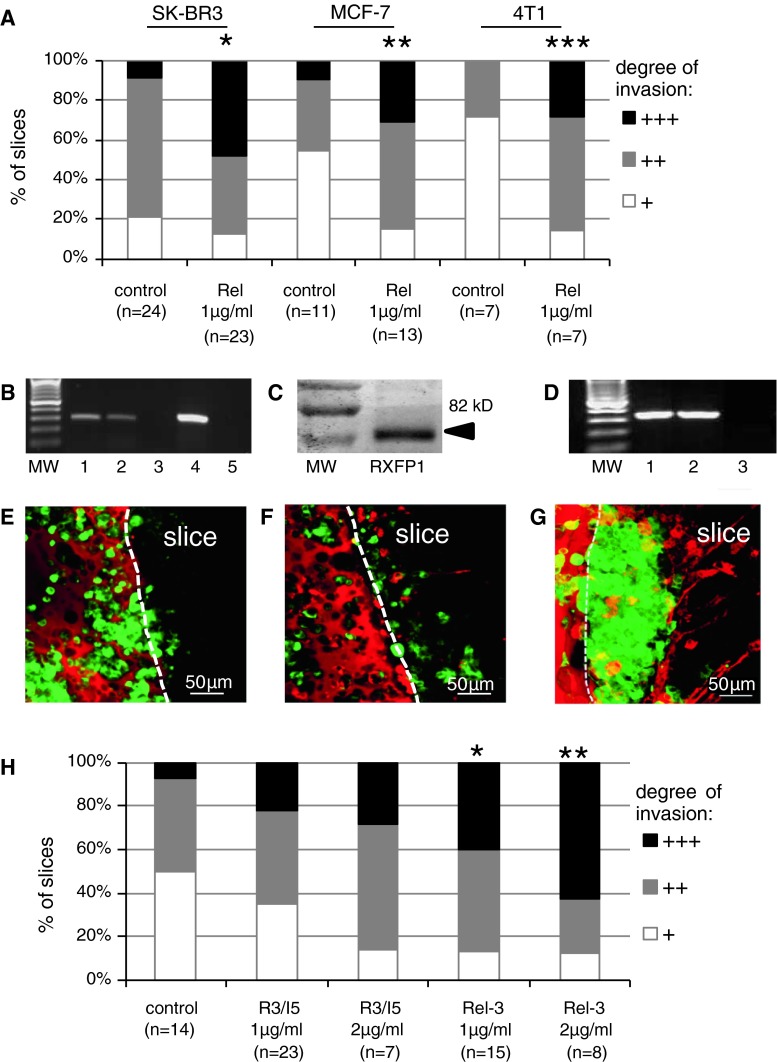
Relaxins enhance brain colonization in whole brain organotypic slice cocultures. **a** Quantification of cancer cell invasion in murine brain slices. The histogram of the different degrees of invasion (for details see “Materials and methods” section) shows enhanced colonization by human and murine breast cancer cells upon incubation with porcine relaxin. *p = 0.012, **p = 0.042, ***p = 0.026, each versus the respective controls. **b** RXFP expression in MCF-7 cells (RT-PCR): *lane* *1* RXFP1 (297 bp), *lane* *2* RXFP2 (302 bp), *lane* *3* RXFP3 (189 bp), *lane* *4* loading control 26S (326 bp), *lane 5* neg. control. **c** RXFP1 protein in MCF-7 cells (Western blot). **d** RXFPs in SK-BR3 cells (RT-PCR): *lane* *1* RXFP1, *lane* *2* RXFP2, *lane* RXFP3. **e–g** Confocal microscopy of whole brain organotypic slice cocultures: *green* GFP-transfected MCF-7 cells; *red* microglia stained with isolectin IB4. Tumour cells on the *left side*, embedded in ECM (showing some unspecific diffuse red staining), invade into the brain slice (*right side*, slice border *dashed line*) and colocalize with red microglial cells. **e** Control, showing only few invading cells and no microglial reaction after 72 h. **f** The RXFP3 agonist R3/I5 (2 μg/ml) does not significantly enhance invasion. **g** Pronounced invasion and microglia accumulation upon addition of relaxin-3 (2 μg/ml). **h** Quantification of MCF-7 invasion in the whole brain organotypic slice cocultures presented in **e**–**g**. *p = 0.011 versus control, **p = 0.013 versus control. (Color figure online)

To mirror the conditions in the animal model and to achieve a purely murine setting, we first used the mouse breast cancer cell line 4T1. As shown in Fig. 3a, porcine relaxin significantly enhanced invasion of the tumour cells into the brain slice. The same could be shown for the human erbB2-positive breast cancer cell line SK-BR3 as well as for the erbB2-negative and hormone receptor-positive cell line MCF-7. In this model, we have previously demonstrated that successful tumour cell invasion requires interaction with microglia which are attracted, activated and subsequently mediate the transport of the malignant cells into the brain tissue [19]. Addition of relaxin clearly enhanced this reaction and lead to increased accumulation of microglia at the site of tumour cell entry.

Next, we were interested in whether the brain-specific relaxin-3 would achieve the same effect. The genuine receptor for relaxin-3 is RXFP3, expressed predominantly in brain tissue, in particular in the basal regions [29] where the whole brain slices are cut from. Relaxin-3 can additionally bind to RXFP1, also present in the brain. In the tumour cells, we could detect only RXFP1 and 2, RXFP3 was absent (Fig. 3b–d, not shown for 4T1). Invasion of MCF-7 cells as well as microglia accumulation was significantly increased by synthetic human relaxin-3 in a concentration-dependent way (Fig. 3e–h). We then asked whether relaxin-3 exerts its effect via its specific receptor RXFP3 or via RXFP1 in both tumour cells and brain. In contrast to relaxin-3, the specific RXFP3 agonist R3/I5 was only weakly pro-invasive (Fig. 3h), suggesting that relaxin-3 enhances invasion predominantly via RXFP1.

## Discussion

While there is increasing evidence of a tumour-promoting role of relaxins in prostate and several other cancers, the data for breast cancer are still contradictory. Here we show that porcine relaxin significantly enhances growth of breast cancers which had developed spontaneously in an erbB2-overexpressing mouse model. This was associated with a significantly higher proliferation rate in tumours from relaxin-treated animals as well as with upregulation of RXFP1 expression. Additionally, relaxin-treated animals had significantly higher serum levels of E2 and P4, which was accompanied by increased expression of the respective receptors in the tumours. Induction of RXFP1 either directly by its ligand or indirectly via E2 which, in turn, can be upregulated by relaxin [30]), has already been demonstrated in the marmoset monkey [30] and in the pig [31]. In the mouse cervix, activation of ERα-signaling was necessary to enable the proliferative effect of relaxin [32]. This suggests that in our model relaxin stimulates tumour growth by one or both of the following options: either directly via upregulation of its own receptor or indirectly via induction of sex hormones which then enhance proliferation through their corresponding receptors.

As an indication that relaxin did not only act on tumour but also on stromal cells, we found significantly elevated amounts of infiltrating TAM in tumours from relaxin-treated animals. Although the TAM were localized predominantly in the stromal compartment, they also infiltrated into the tumour tissue itself. This demonstrates that interaction between tumour and stroma in this spontaneous model could occur freely without interference of local obstacles, such as formation of a surrounding fibrous capsule, or of species barriers as often the case in artificially induced cancers in both immunocompetent and deficient mice [5, 12, 13]. Since high amounts of TAM are well-known to confer an unfavourable clinical outcome [33], relaxin-induced TAM infiltration may have additionally contributed to enhanced tumour growth.

These data are consistent with our earlier findings that both porcine relaxin and human relaxin-2 enhance breast cancer cell invasion [15, 16]. In vivo, we could show that elevated relaxin-2 serum levels in breast cancer patients correlate with metastatic disease [17] and high RXFP1 mRNA levels are an independent marker of metastasis and shortened survival in dogs [34]. In contrast to these results, other authors described reduced growth of relaxin-2-overexpressing MDA-MB 231 cells [13] as well as a differentiating effect of relaxin on MCF-7 cells in nude mice [12]. Both groups used xenograft models in immunodeficient animals with all the potential problems regarding the microenvironment interaction mentioned above. Additionally, the MDA-MB 231 transfectants produced up to 50-fold enhanced local relaxin-2 concentrations [13]. This is clearly higher than in our model where systemic relaxin levels were around 10^−10^ M and only about fivefold higher in relaxin-treated animals than in controls. The amount of relaxin is an important variable, since Sacchi et al. [14] have shown that MCF-7 cells respond to higher versus lower relaxin concentrations with opposite functional outcome, levels of <8 × 10^−10^ M inducing tumour cell proliferation.

The biphasic concentration effect may also explain the divergent data of another group [35, 36] which generated high intratumoural concentrations of relaxin either by direct overexpression of relaxin in the injected breast cancer cells or by delivery via infiltrating monocytic cells with artificial relaxin overexpression. Although there was excessive matrix remodeling around the tumours which usually is a hallmark of cancer progression, the tumours grew slower than the controls. This surprising effect was attributed to facilitated accessibility for lymphocytes and macrophages with antitumour activity. Supposedly, local relaxin levels were rather high in this model, RLN1 mRNA expression being described as more than fivefold increased in the monocyte approach and 50–180 fold in the transfected tumour cells. Since high concentrations of relaxin have been described to induce a cytotoxic TH1-shift in CD4 + T cells [37], this would in fact support the concept of a T-cell induced antitumour response in this model. However, in vivo tumour-infiltrating T-cells and macrophages usually display a tumour-promoting TH2 and M2 phenotype. Figueiredo et al. [21] have shown that concentrations of relaxin-2 as low as 0.02 ng/ml shift the macrophage phenotype from M1 to M2. Consistently, in our model with relatively low relaxin levels, enhanced infiltration by TAM upon relaxin treatment was not associated with inhibition of tumour growth but with the opposite.

Until now the influence of relaxins on spontaneous metastasis formation is unclear. Since we could not detect any metastases during the life span of our mice, we addressed this question in a brain slice coculture model [19]. There we had shown before that tumour cells need the active help of microglia, the resident macrophages of the brain, for successful invasion. First, microglia is attracted and accumulates at the site of the potential tumour cell entry. Activated microglia then serve as vehicles and guidance structures for the malignant cells into the brain. Porcine relaxin increased microglia accumulation and invasion of each of the tumour cell lines investigated in this model, independent of the species of origin as well as of erbB2 and hormone receptor expression. The effect was dose-dependent and became most significant in higher concentrations. These concentrations are higher than those used in our previous Boyden chamber experiments [15], however, since relaxin is distributed via diffusion within the brain slice, they are necessary to ensure sufficient penetration. To further characterize the receptors which mediate the pro-invasive effect, we applied the brain-specific relaxin-3 which binds with high affinity to its primary receptor RXFP3 but can also interact with lower affinity with RXFP1. Both receptors were present in the brain, but only RXFP1 was expressed on the tumour cells. Synthetic human relaxin-3 induced a similar pro-invasive effect as porcine relaxin. In comparison, the specific RXFP3-agonist R3/I5 did not significantly enhance invasion. Thus, it can be assumed that most of the pro-invasive effect is mediated by RXFP1 both on tumour cells and their stromal recipients, the microglia.

These results are consistent with a recent clinical observation in a patient with hormone receptor-negative breast cancer [38]. There, we found an unusually fulminant progression from a single small osteolytic lesion to widely disseminated metastases in multiple organs during hormonal treatment for oocyte harvest before the planned chemotherapy. This was associated with induction of high relaxin-2 serum levels, strongly suggesting a tumour-promoting effect of relaxin-2 either directly on the tumour cells or via the microenvironment.

Taken together, in a mouse model of spontaneous erbB2-positive breast cancer, where interactions between tumour cells and the stromal compartment are not hampered by species barriers and immunodeficiency, relaxins clearly promote tumour progression. This is obviously due to effects of relaxins on the tumour cells but also on the microenvironment, in particular, the macrophages. We also demonstrate that relaxins can foster metastatic colonization of the brain, again, via direct action on the tumour cells but also on the recipient tissue. These findings strongly argue in favour of a tumour-promoting role for relaxins in breast cancer. Given the context-dependency of relaxin action, further experiments with special attention to an intact microenvironment and to relaxin dosage are warranted.

## Electronic supplementary material

Below is the link to the electronic supplementary material.

Supplementary material 1 (PDF 275 kb)
