# Supplementary material for: Relaxins enhance growth of spontaneous murine breast cancers as well as metastatic colonization of the brain
Source: Clin Exp Metastasis. 2013 Aug 21;31(1):57–65. doi: 10.1007/s10585-013-9609-2 (PMC3892110; doi:10.1007/s10585-013-9609-2)
Supplement: Supplementary file 1 — Supplementary material 1 (PDF 275 kb) [file 10585_2013_9609_MOESM1_ESM.pdf]

Suppl. Figure 1

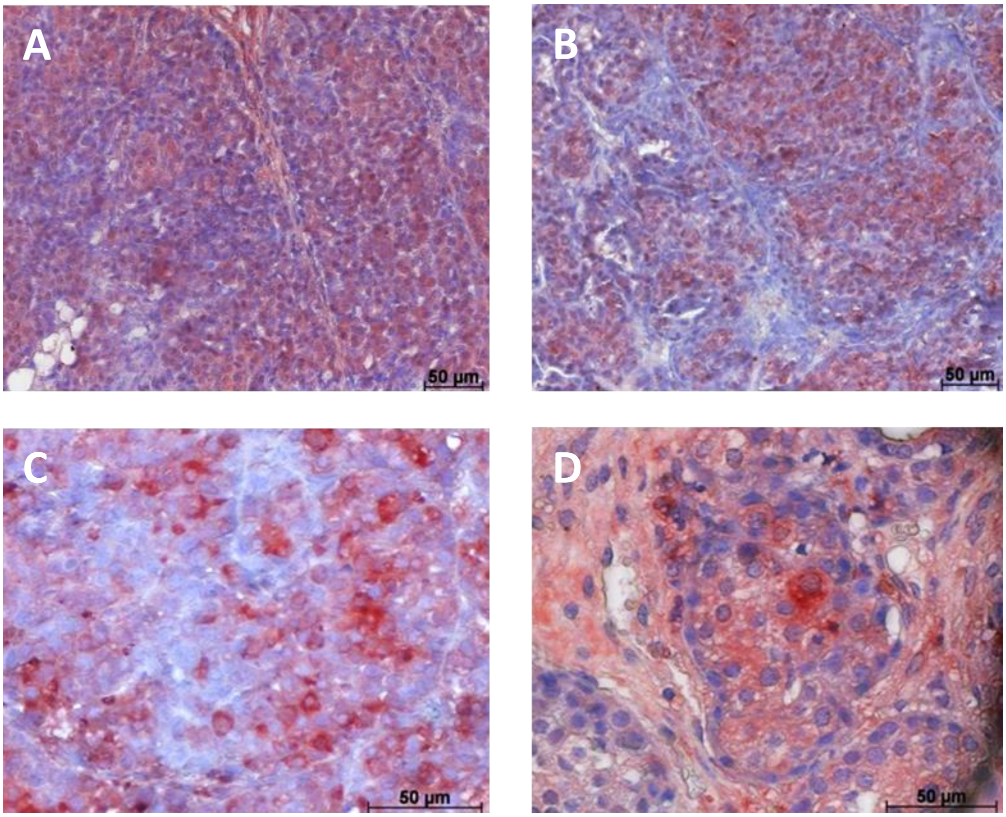

Immunohistochemical detection of relaxin expression in Tg(MMTV-erbB2) mouse breast cancers showing focal as well as diffuse positivity of the tumour cells without any difference between controls and relaxin-treated animals:

A, C = controls

B, D = + relaxin

A, B = magnification 20x

C, D = magnification 40x
